# Acceptability and feasibility of short message service to improve ART medication adherence among people living with HIV/AIDS receiving antiretroviral treatment at Adama hospital medical college, Central Ethiopia

**DOI:** 10.1186/s12889-019-7687-z

**Published:** 2019-10-21

**Authors:** Tamrat Endebu, Alem Deksisa, Warku Dugasa, Ermiyas Mulu, Tilahun Bogale

**Affiliations:** 1 Department of Public Health, Adama Hospital and Medical College, Adama, Ethiopia; 2 Department of Public Health, College of Medicine & Health Sciences, Ambo University, Ambo, Ethiopia

**Keywords:** Acceptability, Short service message, People living with HIV/AIDS

## Abstract

**Background:**

People living with HIV/AIDS are facing sub-optimal adherence to antiretroviral therapy. Short message service innovative strategies have been recommended by the national strategy to support medication adherence among HIV positive people. Thus, this study was conducted to examine the feasibility and acceptability of a short message service to improve medication adherence among people living with HIV/AIDS receiving Antiretroviral Treatment.

**Methods:**

We conducted a cross-sectional survey, from February 5 to 30, 2018, among 422 randomly selected adults living with HIV/AIDS receiving antiretroviral treatment at Adama Hospital. Interviewer administered structured questionnaire was used to collect quantitative data on the feasibility and acceptability of short message services, socio-demographic and clinical characteristics of participants. Qualitative data were also collected from two focus groups to supplement the quantitative findings. Logistic regression analysis was performed to identify factors associated with the feasibility and acceptability of short message services.

**Results:**

Of 420 participants responded to our questionnaire, about nine of ten patients (93.8%) possessed had a mobile phone. Most of the patients (90.9%) were willing to accept SMS to improve their medication adherence. Patients who were in young age, early adult, disclosed their HIV status, having cell phone always and believe short message service aid adherence were more likely to accept short messages on adherence. On the other hand, frequent ART Clinic visit and perceived low confidentiality of short message service were negatively associated with acceptability of short message service.

**Conclusion:**

The acceptability of short message service on adherence to antiretroviral therapy was high among people living with HIV/AIDS central Ethiopia. Authors recommend further studies, piloting or experimenting, that validate the acceptability, feasibility, effectiveness, and scalability of the intervention.

## Background

The advance and rapid expansion of antiretroviral therapy (ART) to tackle HIV/AIDS is one of the most remarkable achievements in global public health history. However, HIV/AIDS remained a major global public health problem. Sub-Saharan countries share unfairly high, about two-third, global HIV/AIDS disease burden [1]. HIV/AIDS remains social, health, and economic menace for Ethiopia. According to Ethiopian Public Health Institution (EPHI) 2017 estimation, about 722,248 people living with HIV/AIDS (PLWH), 391,844 people were on antiretroviral treatment (ART) and 14,872 people died due to the disease at a national level [2].

World Health Organization (WHO) recommends that ART should be initiated in all adults living with HIV, regardless of WHO clinical stage and at any CD4 cell count. Access to ART has been also increasing with time, and this expansion has helped the program reach millions of people living with HIV/AIDS (PLWHIV) [3]. Higher adherence levels to a treatment plan, take medication at prescribed times & frequencies and follow restrictions regarding food and other medications are crucial for better clinical outcomes, including fewer hospitalizations, fewer opportunistic infections, and complete suppression rates. Ensuring clients taking equal or greater than 95% of prescribed doses, prevent the development of resistant to Human Immune-deficiency Virus (HIV), and prevent HIV treatment failure are the goals of adherence to antiretroviral therapy [4].

Studies conducted in different parts of Ethiopia revealed sub-optimal adherence to ART that might result in incomplete viral suppression, the emergence of resistant viruses, increased risk of morbidity and mortality, treatment failure and compromise future therapeutic options. Main barriers to ART adherence included fear of disclosure, forgetfulness, or missing doses, health illiteracy, substance abuse, complicated regimens, missing an appointment to visit clinic and patients being away from their medications [5–7]. Lack of strong retention and patient follow-up mechanisms is also another challenge that hindering to attain the country strategic plan objectives and targets to end AIDS by 2030 [5–10].

Crilly JF.et al. suggest that electronic technologies must be more broadly and effectively implemented to realize their potential to improve health outcomes for vulnerable populations, lower costs, and reduce health disparities [11]. Mobile technology is one of the electronic technologies widely applied in the public health system. Especially, SMS is an efficient and cost-effective, and the infrastructure for SMS is available throughout most of the world [12–15]. The expansion of mobile technology in twenty-first century enable several researchers to thought and examine the potential use of supportive and reminder Short Message Service (SMS) intervention in improving adherence to ART. The low cost and relative ease of sending SMS to mobile phones make it an attractive intervention for supporting ART adherence by using the highly growing mobile phone technology [16–19].

Ethiopia also has a plan to tackle adherence barriers and succeeding to reach 90–90-90 treatment targets by 2020. The national/regional operational plan has recommended implementing SMS innovative strategies to support ART medication adherence [20]. However, the SMS innovative strategy is not yet implemented as well as a limited study conducted to assess its feasibility and acceptability. Thus, we conducted this study to examine the feasibility and acceptability of SMS to improve ART adherence and exploring associated factors among HIV positive people receiving ART in central Ethiopia.

## Methods

### Study setting and design

We conducted an institutional-based survey from February 5 to 30, 2018 among 422 adults living with HIV/AIDS receiving ART at Adama Referral Hospital, central Ethiopia. The hospital located in Adama City, 99 km east of Addis Ababa. The adult prevalence rate of HIV/AIDS in Adama City is 4.6%, which is higher than the urban average prevalence rate (4.2%) of the country. The estimated numbers of PLWH in Adama City were about 25,000 and about half of them (13,500 PLHIV) were receiving ART service.

### Sampling and data collection procedures

We randomly selected 422 PLWH from the list of patients who had an appointment during the study period. Study participants were individually interviewed using structured and pretested questionnaire. The data collection tool had inquiries on socio-demographic and clinical characteristics of the participants, feasibility, and acceptability of SMS on adherence to ART medication. Adherence to ART was measured based on respondents self-report, appointment log and pill count [21].

The English version of the questionnaire translated to Amharic and Afan Oromo (local languages spoken in the City) and back-translated to English to check the consistency. There was three days training for selected data collectors and supervisors on obtaining informed consent and participants’ rights, interview techniques and on the use of the tool. The questionnaire was pretested for clarity, consistency, and reliability. Analysis of the pretest result indicated that our tool to assess acceptability and feasibility had very good reliability (Cronbach’s Alpha = 0.87).

Qualitative data were also collected from two focus groups to supplement the quantitative findings. Eligible participants for the two FGDs were recruited via a purposive sampling method. Sex, age, and position in PLHIV association were considered for segmenting PLWH for the FGDs. The first group, a group of 11PLWH, was purposively formed from PLWH association leaders who receive ART service from Adama Health and Medical College (segmentation based on position in the PLHIV association). These leaders were recruited from 11 PLWH associations found in Adama City. The second group, a group of 9 PLWH, was also purposively formed from members of PLHIV associations based on participation in the PLHIV association, sex, and age categories.

### Data processing and analysis

There was close supervision of the data collection process and questionnaires were filled daily to ensure accuracy of the data. Quantitative data were entred entered into Epi-Info version 7.1.5. Then, the cleaned and edited data were exported to SPSS version 21.0 for analysis. The normality and distribution of data were checked using histograms, plots and statistics. Descriptive findings were summarized using frequencies, percentages, mean, median and standard deviations.

We performed a univariate logistic regression analysis to assess the impact of a number of factors on the outcome variable. The association between each independent variable and the dependent variable (acceptability of SMS) was assessed using univariable logistic regression analysis. The multicollinearity of independent variables was checked using a variance inflation factor (VIF). To control confounding effects (evaluate the consistency of the effect after adjusting other variables), variables significantly associated with the outcome variable (at *p* < 0.25) in the univariable model were entered into the multivariable logistic regression model. Statistical associations were asserted based on 95% CI and two-sided 5% level of significance (α < 0.05).

The text of the focus group discussion was processed using thematic content analysis. The notes and audio-recorded FGDs were familiarized through reviewing, reading and thoroughly listening. Then, the data were transcribed to a word processing document. The transcribed data were translated into English. Selective coding of the transcript was performed based on the major themes,; but emergent categories were also noted. We incorporated relevant quotations from transcripts to illustrate the main themes. Finally, triangulation of different data sources was performed to verify the findings.

## Results

### Socio-demographic and clinical characteristics

Four hundred twenty responded to our questionnaire, a response rate of 99.5%. Majority of the respondents, 242(57.6%) were female. The median age of participants was 35 (Interquartile range: 25–40) years. Most (80.2%) of the participants were urban dwellers. One out of three PLWH had no formal education. The employment rate of respondents was about 75%. However, more than half of them had an average monthly income of less than1000 ETB (about 35 USD). About three-fourth of respondents, 314(74.9%) were living with either spouse or partner or family whereas the remaining were living alone (Table 1).

**Table 1:** Sociodemographic characteristics of study participants (*n* = 420)

Variable	Categories	frequency	Percent
Sex	Male	178	42.4
	Female	242	57.6
Age (year)	15–30	163	38.8
	31–45	209	49.8
	> 45	48	11.4
Marital status	Single	60	14.3
	Married	190	45.2
	Divorced	102	24.3
	Widowed	68	16.2
Education	No formal education	143	34.0
	Primary	122	29.1
	Secondary and above	155	36.9
Employement status	Unemployed	106	25.2
	Employed	314	74.8
Income (ETB)	<=1000	210	53.3
	> 1000	184	46.7
Living condition	Living alone	105	25.1
	Living with spouse/ family/parent	314	74.9
Residence	Urban	337	80.2
	Rural	83	19.8
Owning a mobile phone	Yes	394	93.8
	No	26	6.2

The time at which participants diagnosed for HIV/AIDS and started ART ranged from a few months to more than a decade. About three-quarter of the patients knew their status and most of them started ART treatment before 6 years. The intervals at which about 78% of the patients visit ART Clinic was more than 1 month whereas the remaining were visiting the ART Clinic every month. Majority of participants (85.0%) did not miss their healthcare appointment within the last 12 months while 90.7% of patients adhered to prescribed ART drugs within the last 3 months. More than half of the participants (55.2%) disclosed their HIV status to other than their health care provider (Table 2).

**Table 2:** Clinical and lifestyle characteristics of respondents (*n* = 420)

Characteristics	Categories	Number	Percent
Time since HIV diagnosed	< 6 Years	103	24.5
	6 to 8 years	114	27.1
	9 to11 years	102	24.3
	≥ 12 years	101	24.1
Time since started ART	< 6 years	130	31
	6 to 8 years	121	28.8
	9 to 11 years	95	22.6
	≥ 12 years	74	17.6
Frequency of ART Clinic visit	Every month	91	21.7
	> One Month	329	78.3
Missed healthcare appointment (with in the last 12 months)	Yes	63	15
	No	357	85
Missed to take medication (within the last three months)	Yes	39	9.3
	No	381	90.7
Disclosed HIV status	Yes	232	55.2
	No	188	44.8
substances use (Alcohol, khat, smoking)	Yes	26	6.2
	No	394	93.8

### Feasibility and acceptability of SMS from ART Clinic

Almost all of the respondents, 394(93.8%), owned mobile phone and more than four-fifth of those respondents (84.5%) were having a mobile phone always with them. Participants’ had a different habit of mobile use. Around three-quarters of the respondents (77.7%) reported that there is no time they regularly switch off/out of mobile phone service. About four-fifths of participants (82.5%) did not share their cell phone with other person and one-fifth of participants (18.5%) were using mobile phone password. About 71.8% of the participants were able to read SMS and about 59.9% of them were able to send text messages using their mobile phone.

Most of the patients (91%) were willing to accept SMS on ART treatment from ART Clinic and about 81.5% of them perceived that SMS would aid medication adherence. Nearly two-thirds of the participants were willing to pay for the SMS received based on national telecommunication tariff. Overall, about 62.5% of patients satisfied the criteria set for SMS feasibility i.e., own mobile phone, able to read SMS, willingness to accept SMS from ART Clinic and willing to pay for SMS service they may receive based on existing SMS tariff (Table 3).

**Table 3:** Feasibility and acceptability of SMS on adherence to ART medication (*n* = 394)

Variable	Categories	Frequency	Percent
Having a mobile phone always	Always	333	84.5
	Not always	61	15.5
Using more than one phone numbers	Yes	99	25.1
	No	295	74.9
Ever lost mobile phone	Yes	155	39.3
	No	239	60.7
Regular time switch off /out of mobile phone service	Yes	88	22.3
	No	306	77.7
Using mobile phone password	Yes	73	18.5
	No	321	81.5
Share mobile phone with another person	Yes	69	17.5
	No	325	82.5
Ability to read a message on mobile phone	Yes	283	71.8
	No	111	28.2
Ability to send a message using a mobile phone	Yes	236	59.9
	No	158	40.1
Perceiving the likelihood of SMS seen by others (Perceived confidentiality of SMS)	Likely	143	36.3
	Unlikely	251	63.7
Using internet on a mobile phone	Yes	65	16.5
	No	329	83.5
Believe SMS will improve their adherence	Yes	321	81.5
	No	73	18.5
Willingness to accept SMS from ART Clinic	Yes	358	90.9
	No	36	9.1
Willing to pay for SMS (*n* = 358)	Yes	232	64.8
	No	126	35.2
Satisfied set criteria for SMS feasibility	Yes	203	62.5
	No	122	37.5

**Fig. 1:**
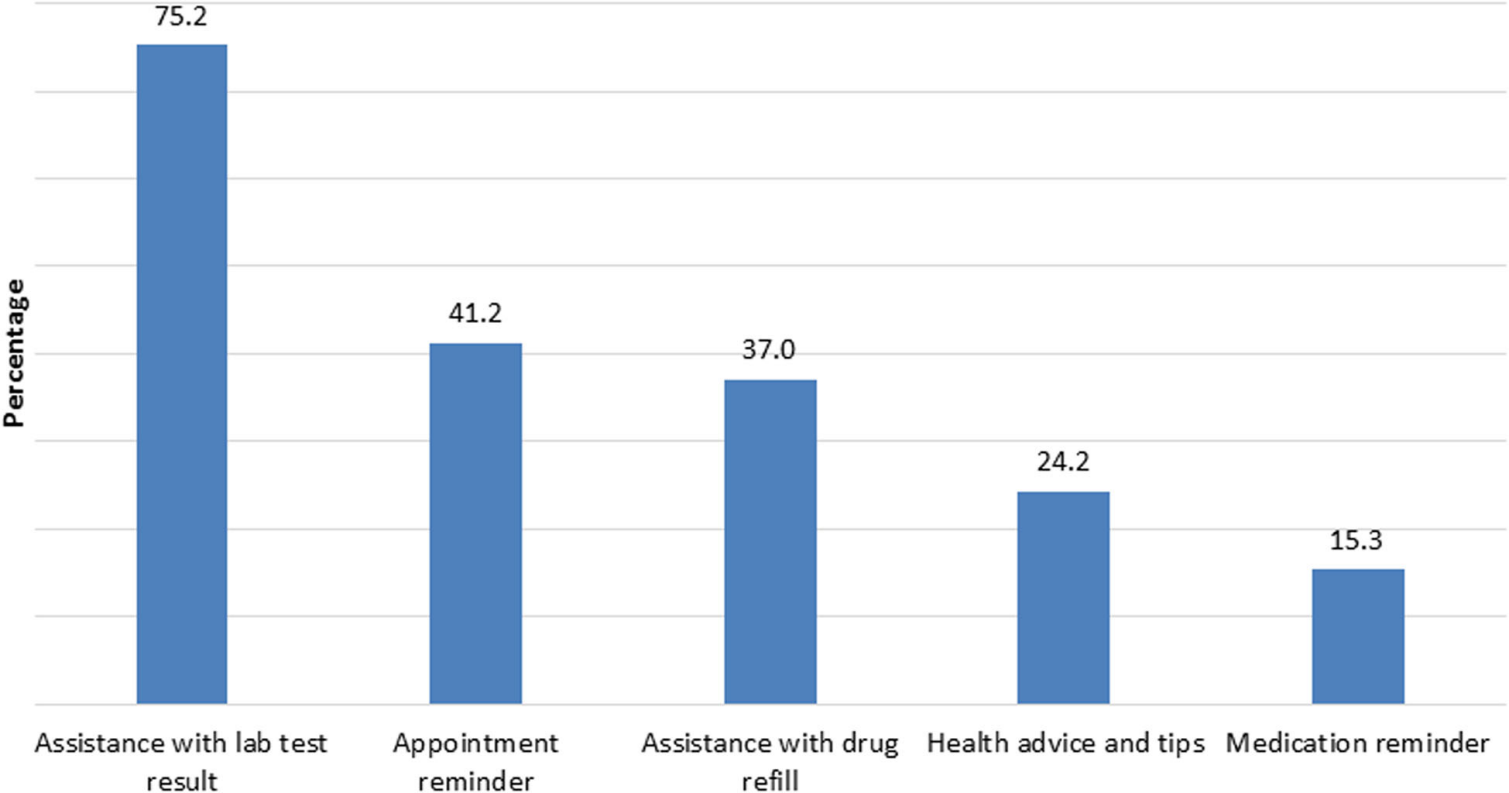
types of SMS that patients on ART would like to have if the ART Clinic starts SMS based intervention

Of those accepting SMS (*n* = 358), three-fourth of respondents, 270 (75.2%) were willing to receive their laboratory test result through SMS. Around two-fifth, 148(41.2%) were willing to receive SMS as Clinic appointment reminder, more than one-third, 133 (37.0%) as drug refill assistance and around one quarter, 87 (24.2%) as health advice/motivational tips and 55(15%) willing to receive SMS as medication reminder (Fig. 1).

### The potential usefulness of short message service and acceptability to support adherence

Two focus group discussions, one with members of PLHIV association (had 11 participants) and one with leaders of PLHIV association (had 9 participants) were conducted. The mean age of the FGD participants was 33.6(±12.8). Out of 20 participants within the two FGD, 11 were male, 8 widowed, 10 had educated secondary level and 18 owned mobile phone (Table 4).

**Table 4:** Socio-demographic characteristics of HIV positive participants involved in FGD (*n* = 20)

Characteristics	Categories	Frequency	Percent
Sex	Male	11	55.0
	Female	9	45.0
Marital status	Single	4	20.0
	Married	2	10.0
	Divorced	5	25.0
	Widowed	8	40.0
Education	No formal education	2	10.0
	Primary	4	20.0
	Secondary	10	50.0
	College and higher	4	20.0
Own Mobile phone	Yes	18	90.0

In these focus group discussions, topics addressed were potential usefulness of SMS, acceptability to support adherence, barriers to use SMS as adherence tool and framing of SMS to support medication adherence. During the discussions, several participants mentioned that the usefulness of SMS and its acceptability to support medication adherence. As participants described:

*“ I think that [short message service] would be something best. Because everybody has a mobile phone today” [Female from FGD PLHIV association member]*

*“I think, communicating via SMS in this time is about modernity. No matter the distance, the place or the time … It (SMS) can reach many people within a second … everybody familiar! For example, people receive and send text messages for holiday wishes, we usually receive messages from many sources, some important others not. so if it (SMS) is designed to support adherence … please welcome” [Male from FGD PLHIV association leaders].*

*“Oh! If it’s so … it’s the best thing I can do for me; most of the time I am confusing as taking my tablet or not on some busy days; usually I used glass on my table … if it put up open … I didn’t take my drug so have to; if the glass put down on the table … already I have taken it” [Female from FGD PLHIV association member].*

*“ … it something good; especially I need it if it will help me reminding me on appointment date” [Male from FGD PLHIV association member].*

*“SMS from ART Clinic is fully acceptable; there are many individuals who interrupt their medication, and schedule of Clinic visit” [Male from FGD PLHIV association leader].*

### Factors associated with acceptability of short message service to improve medication adherence

Bivariate and multivariate analyses were conducted to establish the association between the study variables. At bivariate level sex, age, education, regularity to visit ART Clinic, disclosure status of HIV, having mobile phone always, time regularly switch off/out of mobile phone service, share mobile phone with others, perceived text message confidentiality (likelihood of the received SMS seen by others), ability to read SMS, believed SMS aid their adherence to ART were significantly associated (*p*-value less than 0.05) factors with the acceptability of SMS service at bivariate level. All of the variables with p-value less than 0.05 at bivariate analysis were incorporated in the final multivariable logistic regression model to control the effect of confounding. The Hosmer-Lemeshow goodness-of-fit shows a non-significant Chi squire (Chi-square = 6.331, df = 8, *p*-value = 0.610), which indicated the logistic model was good fit.

In multivariate logistic regression analysis, six variables: age, frequency of visit ART Clinic, disclose HIV status to anyone other than their healthcare provider, having mobile phone at all time, perceiving SMS confidentiality and believe in SMS can aid adherence are factors associated with the acceptability of SMS to improve medication adherence (Table 5).

**Table 5:** Factors associated with the acceptability of SMS to improve medication adherence among patients on ART

Variables	Accept SMS from ART Clinic		Logistic regression		*p-value*
	Yes	No	COR(95%CI)	AOR(95%CI)	
Sex					
Male	162 (94.2)	10 (5.8)	2.15 (1.01–4.59)*	2.04 (0.78–5.22)	0.23
Female	196 (88.3)	26 (11.7)			
Age					
15–30	143 (93.5)	10 (6.5)	3.18 (1.17–8.63)*	6.63 (1.79–24.53)**	0.031
31–45	179 (90.9)	18 (9.1)	2.21 (0.89–5.47)	3.3 (1.07–10.19)**	
> 45	36 (81.8)	8 (18.2)			
Education					
No formal education	110 (85.3)	19 (14.7)	0.32 (0.14–0.76)*	0.59 (0.17–2.06)	
Primary	104 (92)	9 (8)	0.64 (0.24–1.72)	0.82 (0.22–3.07)	0.14
Secon/higher	144 (94.7)	8 (5.3)			
Visit ART Clinic					
Every month	67 (81.7)	15 (18.3)	0.32 (0.16–0.67)*	0.31 (0.12–0.78)**	0.001
> One Month	291 (93.3)	21 (6.7)			
Disclose HIV status					
Yes	206 (94.5)	12 (5.5)	2.71 (1.31–5.59)*	3.15 (1.26–7.89)**	0.014
No	152 (86.4)	24 (13.6)			
Having a mobile phone					
Always	311 (93.4)	22 (6.6)	4.21 (2.02–8.79)*	2.95 (1.03–8.40)**	0.027
Not always	47 (77.0)	14 (23.0)			
Time regularly switch off					
Yes	74 (84.1)	14 (15.9)	0.41 (0.20–0.84)*	0.59 (0.24–1.50)	0.081
No	284 (92.8)	22 (7.2)			
Share mobile phone with others					
Yes	69 (80.2)	17 (19.8)	0.27 (0.13–0.54)*	0.61 (0.23–1.62)	0.29
No	289 (93.8)	19 (6.2)			
Perceived SMS confidentiality					
Low	114 (79.7)	29 (20.3)	0.11 (0.05–0.27)*	0.30 (0.11–0.86)**	0.005
High	244 (97.2)	7 (2.8)			
Ability to read SMS					
Yes	270 (95.4)	13 (4.6)	5.43 (2.64–11.17)*	1.57 (0.49–4.96)	0.151
No	88 (79.3)	23 (20.7)			
Believe SMS aid adherence					
Yes	304 (94.7)	17 (5.3)	6.29 (3.08–12.87)*	4.36 (1.59–11.97)**	0.019
No	54 (74)	19 (26)			

* = significant at p < 0.05 at uniariable level, ** = significant at p < 0.05 at multivariable level

Odds of accept SMS from ART Clinic among participants from the age group of 15–30 years were 6.63 times more likely as compared to those age group of greater than 45 years of age [AOR = 6.63, 95% CI (1.79–24.53)]. Respondents from the age group of 31–45 years had 3.3 times higher odds of accepting SMS from ART Clinic as compared to those age group of greater than 45 years of age [AOR = 3.3, 95% CI (1.07–10.19)].

Odds of accepting SMS from ART Clinic among those visit ART Clinic every month were 69% less likely as compared to those visit ART Clinic within a time interval of more than 1 month [AOR = 0.31, 95% CI (0.12–0.78)]. Patients disclosed their HIV status were 3.15 times higher odds of accepting SMS from ART Clinic when compared to those did not disclose their status [AOR = 3.15, 95% CI (1.26–7.89)]. Respondents, having cell phone always were 2.95 times more likely to accept short message service as compared to those having a cell phone not always [AOR = 2.95, 95%CI (1.03–8.40)]. Participants who perceived confidentiality of SMS as low were 70% less likely accept SMS from ART Clinic as compared to those who perceived high confidentiality of SMS [AOR = 0.30, 95%CI (0.11–0.86)]. Participants who believe SMS aid adherence were 4.36 time more likely accept SMS from ART Clinic as compared to those do not believe SMS aid adherence [AOR = 4.36, 95%CI (1.59–11.97)] (Table 5).

### Perceived barriers to use SMS to improve ART adherence

During FGD, participants identified two main challenges to SMS from the ART Clinic. Several participants mentioned that mobile phone user privacy and confidentiality of short text message as a serious issue (Table 6). Sharing mobile phone with families and keeping a personal mobile phone in a place where others could access were common practices. The following were described quote:

*“I am widowed and have 5 children. Only two of them know my HIV status, so when I take my phone to charge, someone might read the messages” [Female from FGD PLHIV association member]*

*“I think! Hard to accept such thing [SMS] … my phone is not the only mine, but it also belongs to my friends too … we share everything each other … . But nobody knows my HIV status” [Male from FGD PLHIV association member]*

*“My fear is that one may ask you for your phone and when you give it … they may read your messages and begin dissemination gossips about you” [Male from FGD PLHIV association member]*

*“I might be with somebody, who doesn’t know my status … if I so received SMS it may cause me unintentional disclose of my status” [Male from FGD PLHIV association member]*

*“I could not think it (SMS) is acceptable to me … . I have not yet disclosed my status even for my husband … so receiving SMS might be exposed my secrete” [Female from FGD PLHIV association member]*

*“No matter with many families of HIV positive individuals … . I scared that still there high stigma and discrimination among our community” [Male from FGD PLHIV association leader)*

**Table 6:** Categories and Codes identified in the qualitative analysis

Categories	Code	Frequency of mentioned	Percent
The potential usefulness of SMS	Accessibility	10	50%
	Familiarity with SMS	3	15%
	Perceived benefit	6	30%
Acceptability to support adherence	Acceptance	11	55%
	Ease of use / Convenience /	2	10%
	Self-efficacy	1	5%
	Disclosure of HIV status	5	25%
Barriers to use SMS as adherence	Confidentiality	14	70%
	Lack of education/ Mobile phone literacy	4	20%
	Availability/Access	3	15%
	Affordability	3	15%
	Stigma and discrimination	5	25%
Suggesting/framing of SMS	Confidentiality of messages	9	45%
	Personalization of messages	7	35%

### Suggestions for SMS confidentiality issues

Participants in both groups argued for coded messages that would help maintain confidentiality. SMS from ART Clinic would be best if the messages did not mention the words like ‘HIV’ or ‘Hospital’ or ‘drug’ so as not to compromise the aim of the message content. Participants point out that it would be better to have pre-informed and agreed on the code of the message. The following were described quote:

*“If we are ready to accept the idea … it doesn’t matter if we received SMS that not mention the word like hospital or ART or HIV... Instead of that it might be possible to use a code such as … for example June 20 (Friday) this might be the appointment date reminding system”. [Male from FGD PLHIV association leader]*

*“If you send me SMS for example: ‘Time to drink your tea’ I can understand that it reminds me … ‘time to take my pill’. [Male from FGD PLHIV association leader]*

## Discussion

Key findings from this study showed, high accessibility of mobile phone and acceptability of SMS from ART Clinic. Age, frequency of visit ART Clinic, disclose HIV status to anyone other than their healthcare provider, having a mobile phone at all time, perceiving SMS confidentiality and believe in SMS can aid adherence are factors associated with the acceptability of SMS to improve medication adherence.

This study finding has shown high accessibility of mobile phone which make sending SMS from ART Clinics feasible. The qualitative data of this study also supports this finding. Studies carried out in different settings, South Africa [22], Peru [23] and United States [24], reported similar finding to the current study. But, the result from this study was higher than the findings from Uganda [12, 25] and North West Ethiopia [26]. The differences might be due to the study settings and the advances in information and communication technology (ICT) infrastructure.

Despite the high acceptability, the feasibility of intervention (SMS to improve adherence) seems limited by the considerable high number of illiteracy among our study participants. Our finding shows that around two-thirds of patients were able to read a short text message on their mobile phone. This finding is almost the same with a similar study conducted in North West Ethiopia at University of Gondar Hospital (72.2%) [26]. But, it is lower than similar studies conducted in Uganda and South Africa [12, 22]. The disparity might be due to the literacy rate and economic status of the countries. Significant numbers of illiterate study participants’ were willing to receive SMS from the ART Clinic. We speculate, although the participants do not understand the content of the message, they intend to use as an alarm to take ART medication or obtain assist from people around them.

Around nine of ten of patients were willing to accept SMS from ART Clinic. The qualitative result of this study also supports this finding. Two studies from South Africa reported figures that support our finding 88.1 and 96% [13, 22]. Studies conducted in Chine and North West Ethiopia published a lower proportion of patients than ours were willing to accept SMS from ART Clinic [18, 26]. This discrepancy might be due to the difference in the fast-growing mobile phone technology, the need for multipurpose of mobile phone and better understanding level of the usefulness of SMS based intervention. In this study it is also shown, respondents perceived SMS can support their ART adherence are more likely to accept as compared to those do not perceive SMS can support their adherence to ART.

Different studies revealed that the age of the patient is correlated with the acceptability of SMS, better among younger PLWH than older counterparts [18, 26]. The finding of our study supports this fact. The acceptability of SMS was significantly low among elder adults (greater than 45 years). This might be due to the early adopters and better understanding of the technology among the young age generation.

Respondents who visit ART Clinic more frequently were less more likely to accept SMS from ART Clinic compared to those visits ART Clinic within a time interval of more than one month. This is supported by a study from Peru [23]. We would like to argue that some busy patients or those could not visit Clinic as per the appointment may need the kind of intervention they easily accessed.

Patients disclosed their HIV status were more likely to accept SMS from ART Clinic when compared to those did not disclose their status. The qualitative finding also supported this finding. As one female patient stated *“I could not think it (SMS) is acceptable to me …*. *I have not yet disclosed my status even for my husband … so receiving SMS might be exposed my secret”.* The other male patient stated *“my fear is that one may ask for your phone and when you give it … they may read your messages and begin dissemination gossips about you”.* This is in line with a study in Uganda, South Africa, and Kenya [22, 25, 27]. This is might be the fact that still there is high stigma and discrimination among the community.

Patients who perceived low confidentiality of SMS were also less likely to accept SMS from ART Clinic as compared to those perceived high confidentiality of SMS. Qualitative data results also support this finding. Confidentiality of SMS was mentioned several times during the FGD as a challenge to accept the SMS-based intervention. Sharing mobile phone with family members and friends was found to be a challenge since text messages can disclose HIV-status. One young male stated *“I think! Hard to accept such thing [SMS] … my phone is not only mined, but it also belongs to my friends too … we share everything each other … .but nobody knows my HIV status”.* This finding is similar to a study from Uganda and South Africa [22, 25]. This may be due to low HIV status disclose and fear of stigma and discrimination. For these challenges, participants recommended SMS from ART Clinic would be best if the messages did not mention the words like ‘HIV’ or ‘Hospital’ or ‘drug’ to maintain confidentiality.

The likelihood of accepting SMS from ART Clinic was higher among patients who perceived SMS can support their ART adherence than those do not perceive SMS can support their adherence. The qualitative finding of this study also supports this finding. One female stated, *“Ok … I think that [short message service] would be something best. Because everybody has a mobile phone today”.* The result is similar to qualitative studies conducted in Uganda and Chine [12, 18]. This result might explained by the patients’ level of education/literacy and understanding the service.

## Conclusion

Most of our participants own mobile phone and the acceptability of short message service to improve ART adherence was high among patients receiving ART in Central Ethiopia. However, the feasibility of SMS is limited by the significant number of illiteracy among respondents. Age, frequency of visit ART Clinic, HIV disclosure status, ownership of a mobile phone at all time, SMS perceived confidentiality and benefit were the factors associated with the acceptability of SMS to improve medication adherence.

Authors recommend that future research on the subject should focus on practical piloting or experimenting of the acceptability, feasibility, and effectiveness of the intervention. Any interested initiators of SMS based intervention shall consider age, protect the confidentiality of the text message by using mechanisms such as message code, avoiding some indicative words like HIV, ART, and other forms of SMS prior to development and implementation of an intervention. On top of this, we suppose awareness creation, demonstrating clear benefits and use of SMS for the ART patients will enhance the intervention.

## Data Availability

The datasets analyzed during the current study are available on the hand of the corresponding author. However, we feel sorry for unable to put in the repository as we did not have consent from participants to disclose individual findings.

## Abbreviations

ART: Antiretroviral Therapy
EPHI: Ethiopian Public Health Institution
FGD: Focus Group Discussion
HIV/AIDS: Human Immune Virus/Acquired Immune Deficiency Syndrome
PLWH: People Living with HIV/AIDS
SMS: Short Message Services
VIF: Variance Inflation Factor
WHO: World Health Organization

## References

[1] UNAIDS, Global-AIDS-update-2016_en.pdf. Accessed on 2017 September at https://www.unaids.org/sites/default/files/media_asset/global-AIDS-update-2016_en.pdf.

[2] ETHIOPIANS AND AMERICANS IN PARTNERSHIP TO FIGHT HIV/AIDS, Differentiated Care in Ethiopia - The Way Forward_FINAL.pdf PEPFAR, March 2017. Available at http://www.differentiatedcare.org/Portals/0/adam/Content/qwdngVqYIUqSl_wl-tG2zQ/File/Differentiated%20Care%20in%20Ethiopia%20-%20The%20Way%20Forward_FINAL.pdf

[3] World Health Organization. Consolidated Guidelines on the Use of Antiretroviral Drugs for Treating and Preventing HIV Infection: Recommendations for a Public Health Approach. [Internet]. Accessed on 2017 September 12 from https://apps.who.int/iris/handle/10665/208825.

[4] World Health Organization. Adherence to long-term therapies: evidence for action. 2003, Geneva 27, Switzerland. Accessed on 2017 September 10 from https://apps.who.int/iris/bitstream/handle/10665/42682/9241545992.pdf;jsessionid=B05279F40BFB86FD69E41727A1D55193?sequence=1.

[5] Reda AA, Biadgilign S (2012). Determinants of adherence to antiretroviral therapy among HIV-infected patients in Africa. AIDS Res Treat.

[6] Asmare M, Aychiluhem M, Ayana M, Jara D (2014). Level of ART adherence and associated factors among HIV Sero- positive adult on highly active antiretroviral therapy in Debre Markos referral hospital, Northwest Ethiopia. J Antivir Antiretrovir.

[7] Mitiku H, Abdosh T, Teklemariam Z (2013). Factors affecting adherence to antiretroviral treatment in Harari National Regional State. Eastern Ethiopia ISRN AIDS.

[8] Markos E, Worku A, Davey G. Adherence to ART in PLWHA and Yirgalem Hospital, South Ethiopia. Ethiop J Health Dev [Internet]. 2009; 22(2) 174.

[9] Gesesew HA, Ward P, Hajito KW, Feyissa GT, Mohammadi L, Mwanri L (2017). Discontinuation from antiretroviral therapy: a continuing challenge among adults in HIV Care in Ethiopia: a systematic review and meta-analysis. PLoS One.

[10] Shewangizaw Z, Ketema A. Assessment of adherence to highly active antiretroviral therapy and associated factors among people living with HIV at Debrebrihan Referral Hospital and Health Center, Northeast Ethiopia: a cross-sectional study. HIVAIDS - Res Palliat Care. 2015 Mar; 75. 10.2147/HIV.S79328 PMC4362904 25792856

[11] Crilly John F., Keefe Robert H., Volpe Fred (2011). Use of Electronic Technologies to Promote Community and Personal Health for Individuals Unconnected to Health Care Systems. American Journal of Public Health.

[12] Kim Jiho, Zhang Wendy, Nyonyitono Maureen, Lourenco Lillian, Nanfuka Mastula, Okoboi Stephen, Birungi Josephine, Lester Richard T, Kaleebu Pontiano, Munderi Paula, Moore David M (2015). Feasibility and acceptability of mobile phone short message service as a support for patients receiving antiretroviral therapy in rural Uganda: a cross-sectional study. Journal of the International AIDS Society.

[13] Nachega J, Skinner D, Jennings L, Magidson J, Altice F, Burke J, et al. Acceptability and feasibility of mHealth and community-based directly observed antiretroviral therapy to prevent mother-to-child HIV transmission in South African pregnant women under Option B+: an exploratory study. Patient Prefer Adherence. 2016 Apr; 683. 10.2147/PPA.S100002 PMC4854240 27175068

[14] GSMA Intelligence, Global Mobile Trends 2017. Available at https://regmedia.co.uk/2017/09/12/gsma-mobile-trends-2017.pdf

[15] Paul Budde Communication Pty Ltd, Ethiopia - Telecoms, Mobile and Broadband - Statistics and Analyses BuddeComm.html, 2017. Available at https://www.researchandmarkets.com/reports/4418557/ethiopia-telecoms-mobile-and-broadband

[16] Mechael P, Batavia H, Kaonga N, Searle S, Kwan A, Goldberger A, et al. Barriers and gaps affecting mHealth in low and middle-income countries: Policy white paper [Internet]. Columbia University. Earth institute. Center for global health and economic development (CGHED): with mHealth alliance; 2010 [cited 2017 Sep 8].

[17] Hartzler A, Wetter T (2014). Engaging patients through Mobile phones: demonstrator services, Success Factors, and Future Opportunities in Low and Middle-income Countries. Yearb Med Inform.

[18] Ma Qingyan, Tso Lai Sze, Rich Zachary C, Hall Brian J, Beanland Rachel, Li Haochu, Lackey Mellanye, Hu Fengyu, Cai Weiping, Doherty Meg, Tucker Joseph D (2016). Barriers and facilitators of interventions for improving antiretroviral therapy adherence: a systematic review of global qualitative evidence. Journal of the International AIDS Society.

[19] Bangsberg David R. (2008). Preventing HIV Antiretroviral Resistance through Better Monitoring of Treatment Adherence. The Journal of Infectious Diseases.

[20] Pepfar, Ethiopia, Country/Regional Operational Plan (COP/ROP) (2017). Strategic direction summary, April 21, 2017. Accessed from https://www.pepfar.gov/documents/organization/272012.

[21] Chalker J, Andualem T, Gitau L (2010). Measuring adherence to antiretroviral treatment in resource-poor settings: the feasibility of collecting routine data for key indicators. BMC Health Serv Res.

[22] Crankshaw T, Corless IB, Giddy J, Nicholas PK, Eichbaum Q, Butler LM (2010). Exploring the patterns of use and the feasibility of using cellular phones for clinic appointment reminders and adherence messages in an antiretroviral treatment clinic, Durban, South Africa. AIDS Patient Care STDs.

[23] Curioso WH, Quistberg DA, Cabello R, Gozzer E, Garcia PJ, Holmes KK, et al.“It’s time for your life”: How should we remind patients to take medicines using short text messages? In: AMIA 2009 symposium: 2009; USA. 2009. p. 129–33. PMC3105604 21633523

[24] Miller CWT, Himelhoch S (2013). Acceptability of Mobile phone Technology for Medication Adherence Interventions among HIV-positive patients at an Urban Clinic. AIDS Res Treat..

[25] Rana Y, Haberer J, Huang H, Kambugu A, Mukasa B, Thirumurthy H, et al. Short Message Service (SMS)-Based Intervention to Improve Treatment Adherence among HIV-Positive Youth in Uganda: Focus Group Findings. Clark JL, editor. PLOS ONE. 2015 Apr 16; 10(4):e0125187. 10.1371/journal.pone.0125187 PMC4400100 25881059

[26] Kebede M, Zeleke A, Asemahagn M, Fritz F. Willingness to receive text message medication reminders among patients on antiretroviral treatment in North West Ethiopia: A cross-sectional study. BMC Med Inform Decis Mak. 2015;15(65):1-9. 10.1186/s12911-015-0193-z PMC4535252 26268394

[27] Kinyua F, Kiptoo M, Kikuvi G, Mutai J, Meyers AF, Muiruri P (2013). Perceptions of HIV infected patients on the use of cell phone as a tool to support their antiretroviral adherence; a cross-sectional study in a large referral hospital in Kenya. BMC Public Health.

